# Garland Allen’s Last Book Project

**DOI:** 10.1007/s10739-023-09726-8

**Published:** 2023-08-08

**Authors:** Jane Maienschein

**Affiliations:** 1 Center for Biology and Society, Arizona State University, Tempe, AZ USA; 2 Marine Biological Laboratory, Woods Hole, MA USA

In the northern summer of 2022, Garland E. Allen worked to complete his book on the history of twentieth century genetics. He corresponded with colleagues, sought feedback, welcomed suggestions, and reported to me that he really wanted to finish the volume “by the end of the summer” and then “by the end of the year.” Late summer found him in Woods Hole, Massachusetts, enjoying his cottage with his family. He rented his favorite desk at the Marine Biological Laboratory as he had so many summers before, starting in the 1970s. Tucked away at the back of the library stacks, his desk looks out over the Eel Pond, and thousands of journal volumes surround it on the ceiling-high shelves.

Gar loved it there, felt at home there, and really wanted to make progress with his book there. The kind librarians and friends checked in on him often, making sure he had something to drink and ready to help him if needed. By that time, Gar’s health was declining. He shuffled along with his walker, sores on his legs hurt him, and prostate cancer cells circulated through his body. Yet he persisted, eager to make progress with his book. And eager to enjoy his favorite place in his favorite library at his favorite summer retreat, enjoying life with his family. I believe Gar was happy there, looking forward.

Gar was an optimist. He hoped to overcome the health problems, and his mind and spirit remained strong, but his body quit. I promised him that I would help him with the book if he got it into press and it needed more work than he could give, helping with tasks like responding to reviewers, checking footnotes, getting illustration permissions, and so on. He did not, however, finish the book. In many of the last emails he sent me, he commented on health details and even occasionally admitted that he was getting a bit discouraged, but he reported that he remained cautiously optimistic. He still wanted to finish his book, but his body took over.

Since I had agreed to help Gar with the book, when he died on February 10, 2023, his older daughter helped find the full manuscript draft on his computer. I sadly determined that the manuscript was not ready for publication as a book, and with both his daughters Tania and Carin we decided not to proceed with getting the book into print. Gar had meant to go back and revise it all rather extensively after he had sent the manuscript to press, and he had planned a final chapter that he said would summarize and explain the book’s perspective. He did not get to that point. Yet working on the book gave Gar a chance to share with colleagues and friends, to communicate with his large network of those with overlapping interests in various aspects of the project. The manuscript represents a lot of work and contains many ideas, rich details, and an approach on which it is worth reflecting for historiographic reasons. This article offers a chance to begin some of that reflection and points readers to the full manuscript, with details at the end. For now, let’s consider: What is this book? Why was it important to Gar? And how does it fit into the larger corpus of his life’s work?

## What is this Book?

In 1975, Gar published *Life Science in the Twentieth Century* (Allen 1975). The century had not ended yet, but he felt it was time to write a synthetic survey of major themes, especially up to the middle of the century. He framed the book in terms of a move in the various studies of life sciences from natural history and morphology to experimental science and a more coordinated “life science” rather than “life sciences.” Gar saw in this transformation what he called a revolt from morphology. He offered the volume as a kind of introduction to the topic, useful for teaching, rather than a monographic argument for a preferred thesis.

Shortly after the *Life Science* book appeared, Indiana University’s History and Philosophy of Science Department invited Gar as a speaker to discuss his ideas in that book and related work from his continuing dissertation-based research on Thomas Hunt Morgan (Allen 1978). I was a graduate student at Indiana, I listened to his talk, and I joined my fellow students in resisting Gar’s interpretation of what he presented as a Marxist or Kuhnian style revolutionary transformation in biology around 1900. The department’s usual approach was for graduate students to take the speaker to lunch, which we did. With lively discussion, we decided to continue at dinner, for which I seem to recall that we ate spaghetti and drank probably cheap wine at my house. We all talked for hours. Gar never became defensive, he never failed to respect our ideas, and he welcomed the discussion. It was wonderful for us students to be taken seriously by such a modest, kind, and inspiring man. Gar invited us to respond.

As a result, Keith Benson, Ronald Rainger, and I decided to challenge Gar publicly. We organized a session for the 1978 History of Science Society meeting to discuss the book. Then we invited Stephen Jay Gould, our advisor Fred Churchill, and Gar to respond. To our amazement, they all agreed. That was an inspiring experience, and I recall the large audience in an overfilled room. I recall working hard to give a talk rather than read a paper. And I also recall being absolutely terrified by the idea of Gould commenting on my work in public. But Gar made it all ok. He was so welcoming and generous that he somehow made us all feel that we were already respected scholars (Maienschein et al. 1981). Later my student David Magnus at Stanford University took up the challenge, looking at the persistence of natural history in particular, and he received the same welcoming response from Gar (Magnus 1993). In the Introduction to the book manuscript Gar was writing when he died, he looked back at that time and wrote “When *Life Science in the Twentieth Century* first came out, Jane and her graduate student cohort . . . critiqued the book and were right about some of my over-generalizations (for example, my claims about the demise of morphology with the rise of experimental biology). But it was done in such a constructive and friendly manner that it helped clarify my thinking ever since.”^1^

Around 2000, Gar decided to take on the task of updating his *Life Science* book to cover the whole century. After considerable reflection and after consulting with many friends and colleagues, however, he decided that the task was impossible. He could write another synthetic textbook-type book only if he focused his project, choosing a particular area of the rapidly expanding and increasingly diverse work that could be taken as representing life science. Focusing on the history of genetics and eugenics made the most sense, he decided.

In 2008, Gar proposed this book idea to Harvard University Press and obtained a contract. He started working. Yet other things got in the way, as they so often seem to do, and he progressed slowly. He wrote some chapters in 2008, then updated them in 2013 and 2015 when he was apparently teaching a course that used some of the materials. Other chapters came later, and he updated several in the summer of 2022 and even up to late January 2023. He entered the hospital February 5, 2023 with sepsis taking over his body, and he died February 10, 3 days before his 87th birthday. He continued to work absolutely as long as was possible, and maybe even a little longer.

During the process, he clearly enjoyed interactions with many colleagues. In addition to many exchanges in person and by email with me, he especially noted valuable interactions with Marsha Richmond (on early genetics), Kim Kleinman (on a number of topics), Douglas Chalker (on genetics and development), Allan Larson (on many chapters including concerning the evolutionary synthesis), Carl Craver (on mechanism and philosophy in general), Glenn Stone (on agriculture), and Mark Adams (on Russian science). He valued the work of many others as well and was always eager to read more, learn more, and thereby teach more.

Gar initially called the new book “From Little Science to Big Science: A History of Genetics in the Twentieth Century in Its Economic, Social and Technological Context.” By the summer of 2022, he referred to the book as “From Darwin to the Double Helix: The Development of Genetics in its Intellectual, Social and Economic Context, 1880-1980.” He had realized that stopping before the Human Genome Project with all its implications would be easier, hence the ending in 1980. In addition, when Michel Morange’s excellent *The Black Box of Biology: A History of the Molecular Revolution* appeared in 2020, translated by Matthew Cobb, Gar mentioned to several of us that he now saw his book as telling the story of genetics in ways that complemented but did not attempt to repeat what Morange already offers (Morange 2020). Also, the theme of little and big science no longer seemed accurately to capture the changes. By the time he died, he had titled the chapters of his last book as follows:

- Table of Contents
- Introduction
- Chapter 1 Genetics and the Life Sciences in the Twentieth Century: Contexts and Themes
- Chapter 2 Mendel, Darwin and Theories of Heredity Before 1900
- Chapter 3 Mendel and the Context of Scientific Investigation
- Chapter 4 The Excitement and Woes of A New Paradigm: The Rediscovery and Early Reception of Mendel’s Work
- Chapter 5 Mendelian Puzzles and Chromosomal Solutions: The Mendelian-Chromosome Paradigm of Heredity: 1910-1915
- Chapter 6 Articulation of the Mendelian-Chromosome Paradigm: Mapping the Chromosomal Landscape, 1915-1940
- Chapter 7 The Making of a Scientific Field: People, Institutions and The Professionalization of Genetics, 1900-1945
- Chapter 8 Breeding Better Corn and Better Chickens: Genetics and Agriculture, 1900-1945
- Chapter 9 Breeding Better People: Genetics and Eugenics: The Control of Human Evolution, 1900-1945
- Chapter 10 Genetics, Evolution and the “Modern Synthesis”
- Chapter 11 Making Genes Functional I: Genetics and Embryology: Nuclear-Cytoplasmic Interactions and Challenging the Nuclear Monopoly
- Chapter 12 Making Genes Functional II: Genetics, Physiological Genetics and the Evolution of Development
- Chapter 13 Making Genes Functional III: From Developmental and Physiological Genetics to Biochemical Genetics, 1900-1941
- Chapter 14 Genetics and Public Controversies in the Post-War Period: The Mutagenic Effects of Ionizing Radiation, and the Genetics of Racial Differences
- Chapter 15 Genetics on Trial: The Lysenko Controversy, 1920-1965
- Chapter 16 Continuity and Discontinuity in the History of Genetics: The Origin and Development of Molecular Biology, 1930-1980
- Epilogue (never written)
- Bibliography

Gar had begun to realize that many of the earlier chapters that he had written years ago had become out of date, and it was starting to look like a formidable task to keep revising and updating them in light of new scholarly work. In correspondence with several of us, he acknowledged that it was a daunting task. Yet he did not admit that it was impossible and kept at the job. Not surprisingly, the early chapters remain much closer to the themes of his earlier *Life Science* book than some of the later chapters on which he was working in late 2022 and early 2023.

As Gar explained in the latest version of his Introduction from summer 2022: “Genetics served as an exemplar of the means by which biology … transcended its largely descriptive, natural history past to take its place alongside the physical sciences as quantitative and predictive. Just as Darwin’s theory had been a focus for relating many disparate areas of biology in the nineteenth century, so genetics came to serve a similar role in the twentieth.” In his choice of language, the new manuscript more often argues for a “transformation” rather than the “revolution” he had presented before. It also continues to see the life sciences as trying to become like the physical sciences, a theme he had emphasized before.^2^

In that Introduction, Gar also explained that he realized that trying to present all of life science was too gargantuan a task, likely to lead to superficial discussions of too many different things without coherence. Focusing on genetics could get at what he was convinced had become the core. And even more importantly, Gar saw that “the narrower focus could also provide a glimpse into the wider context in which biology as a whole developed in the twentieth century. While genetics was propelled forward by a remarkable series of theoretical and technological advances throughout the century, it was also shaped by economic, social and political forces, without whose influence the field could not have grown as rapidly or penetrated so deeply into the many areas of life science that it did.”^3^

Looking at those extrinsic economic, social, and political forces is the reason Gar really wanted to write this book. He didn’t need another book on his resumé, and he knew just how much hard work writing a book really involves. He did not need to repeat work that he had done before, but he wanted to add a new perspective to his earlier emphasis on the intellectual work of science. He wanted to explore these social and extrinsic factors outside of science that he felt were too often undervalued in understanding genetics, and he wanted to write something synthetic in ways that he felt others were reluctant to do as they concentrated on narrower specialized topics. The extent to which he succeeded is an open question that others can help assess.

## Why was this Book Important to Gar?

For Gar, this book was important for a number of reasons. For one, he was orderly and felt that the flood of separate studies of this-and-that had failed to capture a synthetic picture of life science. He intended his book for a general reader, from biology students to historians of biology looking for a synthetic treatment and on to the educated public.

It was important to Gar to provide materials to help with teaching. Having grown up in Louisville, Kentucky, Gar obtained his Bachelor’s degree in English from the University of Louisville, then continued to Harvard for a Master’s in teaching. He used that degree to carry him to the Northfield Mount Hermon School in western Massachusetts, where he taught high school biology for four years. With his colleague and life-long friend Jeffrey J.W. Baker, Gar wrote a biology textbook *Matter, Energy, and Life*, which appeared first in 1965 as a result of that teaching and went through four editions (Allen and Baker 1965). They added *The Study of Biology* in 1967 along with a laboratory manual, also followed by more editions (Allen and Baker 1967). *A Course in Biology* (Allen and Baker 1968) also had a second edition, *A Study of Botany* with Preston Adams (Allen et al. 1970), then *The Process of Biology: Primary Sources* (Allen and Baker 1970), *Twelve Problems in Biology: Open-ended Experiments for Introductory College Biology* with John Hake (Allen et al. 1971), and *Scientific Process and Social Issues in Biology Education* (Allen and Baker 2001). These were all intended as textbooks for teaching, as well as accessible presentations of the central ideas of the life sciences. Gar reported that he and Baker wrote these textbooks after realizing that the alternatives they had available while teaching at Northfield Mount Hermon considered parts and details but not what he saw as systems, and they were all divided up into little topics. His most recent teaching resource was an online collection offered through Washington University with colleague Allan Larson: *Evolution Since Darwin: Topics in the History of Evolutionary Biology, Primary Sources, 1858-1958* (Allen and Larson 2021).

That is a lot of biology textbook writing. Most historians who know Gar’s books on Morgan and his *Life Science* are probably not even aware of this body of work. Yet his strong commitment to education about science, and his eagerness to produce materials to help both instructors and students see the scientists and the scientific process behind the current “facts” motivated him. It mattered to him to write synthetic books with broad scope to help us see beyond the details in front of our eyes. That desire drove him to take on this attempt to update some themes and replace his early *Life Science* book*.*

As Gar wrote in his drafts of his book, “I have had a fascination with, and proclivity for, synthetic approaches to history. These approaches not only bring in many strands of a story and show their interrelationships, but can also provide key understandings into the causal factors behind historical development. They present the ‘big picture,’ or what we now call the ‘systems approach.’ At the same time I’m also aware that large-scale endeavors also have their potential pitfalls: from oversimplification to picking and choosing only the strands of a story that fit preconceived notions. But these are pitfalls in the pursuit of any historical investigation, though perhaps more prominent in large-scale synthetic works; a sensitivity to these pitfalls can help avoid the most egregious errors. . . it has been a challenge and an immense amount of fun to make the attempt.”^4^

Further in his Introduction chapter, he made two “confessions” about his approach. First, he remained enchanted by Thomas Kuhn’s *The Structure of Scientific Revolutions*, which he had read during his first summer of graduate school. He loved the broad sweeping look at scientific changes over time, as well as emphasis on different ways of looking at the world through scientific paradigms. As Gar’s friend and colleague Carl Craver noted in correspondence with me, Gar had little patience for philosophical objections to the ideas of incommensurability and the relativism many find in Kuhn and preferred to read him more charitably as providing tools for understanding the evolution of biology. Hence, Gar admitted, his attraction to the idea of revolutionary changes in biology.^5^

Second, Gar confessed to a commitment to Marxist materialism. This may be the place where many of us had the most arguments with Gar over the decades, but it is also where Gar was absolutely committed to trying to help us understand his perspective and why it mattered to him. I liked to tease him about his “Harvard Marxism,” which held it acceptable to own several houses and a lovely burgundy Mercedes, but Gar simply smiled and responded that it was perfectly fine until the revolution. For Gar, reading Marx had opened his eyes to social and economic injustice. He reported that he had led a sheltered life until he went to Harvard for his Master’s in teaching and then on for a PhD after his four years teaching high school biology.

His self-described lack of awareness changed at Harvard, when Gar became involved with various political activities. He marched and spoke out in favor of civil rights, against the Vietnam War, and then in 1970 went to Cuba for five months to cut sugar cane with farmers there and learn about their views of socialism. He reminisced that he began seriously to read Marx when he returned, and he saw there a broad, synthetic thinker who viewed the world in systems of systems rather than just a jumble of individual things. Marx’s *A Contribution to the Critique of Political Economy* (Marx 1859) had a particularly profound impact on Gar around 1971 or 1972.^6^ Gar saw Marxist “historical materialism” as a way to discover the causal factors that underlie and enable social, political, and scientific change. In Gar’s interpretation, Marx called for realizing that a dynamic yet constantly evolving system led to changes over time through debate and exchange of ideas; occasionally the process led to revolution. Reading this work and discussing it with others clearly shaped Gar’s thinking in his earlier *Life Science* book. He also saw it as a foundation for his latest book project on the history of genetics.

Gar reported that it was his reading of Marx that led him to realize the importance of agriculture for understanding genetics, as he discusses in his chapter on the genetics of breeding corn and chickens. The technologies involved in producing food depend on fertilizers, equipment, and understanding heredity in order to control and shape it in producing more productive crops. Of course, others have recognized and documented the role of genetics in shaping agriculture, but Gar felt he could go further in showing how the economic imperatives shaped genetics as well. It was this “dialectical” interplay of science and social, economic, and political factors that he sought to capture with his history of twentieth century genetics.^7^

Change, Gar believed that both Kuhn and Marx had shown us, is dynamic and takes place at a systems level, and he felt that the approach would display patterns and uniqueness. He felt that the combined perspective of the two thinkers helped illuminate what happened with genetics during the Cold War, especially in the Soviet Union. Mark Adams has carefully studied Soviet science, and he reports that he had his disagreements with Gar over interpretation while also working to make sure Gar’s story in his new manuscript was accurate.^8^ Yet the two clearly both enjoyed their decades of exchanges, and they were exchanging substantive emails up to just shortly before Gar’s death.

It is especially important here to appreciate what it meant to Gar to bring together his grad school-inspired enthusiasm for the work of Kuhn and Marx in interpreting the history of genetics. And also we must understand that he did this against a background of deep commitment to social justice in ways that he recognized science and technology could disrupt or support. As Gar began to feel that the ongoing book manuscript was getting close to complete, he wrote to me on January 22, 2023 that he felt he absolutely needed to write a new concluding chapter as well as to rethink some parts of the earlier chapters. Apparently, he wrote the same to others. He wanted to make sure the Kuhnian and Marxist analyses were more thoroughly woven throughout the volume, and he wanted to explain why these perspectives make a difference to how we should understand twentieth century genetics. Unfortunately, he did not write that conclusion, and it is difficult to extract the full sense of what he was thinking from the other chapters alone.

While Gar repeatedly emphasized the importance of socio-political and economic contexts, his discussions often remained more traditional in approach, focused on ideas and methods and interactions among those doing the science and the results they produced. In Chapter 1, Gar emphasized that the book would look at the emergence of biology as a more unified field and as one recognized as important alongside physics. He laid out what he saw as the themes for his book:^9^

How Biology Gets Done: From Speculative Theorizing to Experimental Hypothesis-Testing

Who Does Biology: The Transition from Amateur to Professional

Where Biology Gets Done: Changing Institutional Bases for Biology and Genetics

Patronage: From Private Philanthropy to Government Support

The Movement of Capital and the Industrialization of Biology

The Technological Drive and the Rise of Molecular Biology and Genetics

Genetics and Its Social Relations

Genetics and Medicine

The International Character of Genetics

Philosophical Trends in Twentieth-Century Biology

Yet several of us reading the manuscript commented that what looked like Gar intended as organizing themes actually seemed like a disconnected list. He responded that they were not meant to be organizing so much as important factors to which he wanted to point. Instead of mapping neatly onto specific chapters organized around those themes, they tend to pop up in particular places in multiple chapters. They are more nearly items to note in wherever Gar showed how they are relevant. Each theme takes on importance in different places, as part of a different story. His ambition was to illustrate how as science evolves over time, it continuously interacts with its social, economic, and political contexts. There is much for readers to discover in working through Gar’s chapters and reflecting on what he intended as his perspective.

## How Did It Fit into the Larger Corpus of His Life’s Work?

Recognizing the importance of social, political, and economic factors is not new in the history of science. Yet, recall that Gar’s *Life Science* book was published in 1975 and completed before that. At the time, he was just starting to absorb what he saw as Kuhn’s message and just starting to read Marx more seriously. In effect, this second synthetic-type look at life science in the twentieth century was his chance to apply more fully what he saw as his Kuhnian-Marxist approach. Whether it was possible to succeed with that mission is an open question. Gar himself realized that even though he had hoped to have finished the book by the end of summer 2022, he was not very close. He had not yet worked through the Kuhnian and Marxist themes in the way he had intended, which he hoped to be able to do with that substantive last chapter or Epilogue he proposed. Perhaps he was beginning to realize that it was not possible to write such a synthetic conclusion given the complexities and given the continually shifting landscape of other excellent histories of biology and the ways they shed light on aspects of life science in society.

Gar’s study of Morgan had followed scholarly traditions of the day. For his dissertation and then his book on Morgan, he dug deeply into archival collections, read Morgan’s published work, studied relevant historical literature, and engaged other writings about Morgan. He produced a marvelous study of the scientific ideas and the man behind them. That approach simply isn’t possible for textbook-style synthetic works. As Gar and his colleague Jeffrey Baker surely learned with their biology textbooks, it is traditional in such works to build on the work of others. Similarly with Gar’s *Life Science*, he drew on the scholarship of many rather than trying to study each and every detail himself. He always tried to give credit, and he discussed ideas that he found especially original or with which he had disagreements.

This approach becomes nearly impossible as the body of relevant work explodes. Though Gar tried to keep up, he realized that he was losing ground in some areas. This may have deterred him from finalizing the manuscript. I accused him of “perfectionizing” in trying to pursue every possible source. I see now that he was not doing that, but he was working to keep his narrative flow while working in new discoveries. He was trying to grasp how to present what he saw as his original interpretation based on Kuhn and Marx. He could have continued forever.

Gar’s legacy lies with both his close scholarly study of Morgan and with his attempts to provide more sweeping synthetic works that looked more broadly. In doing the latter, he hoped to capture patterns of scientific change and to show how science works. With *Life Science* and with the book manuscript I am describing here, Gar did a tremendous job of pulling together many disparate parts of the study of life science into one story. He drew on many resources from many other historians and some primary sources, and his work is always carefully researched and documented. He did achieve more of a synthesis than other authors have even attempted, and in showing possible interpretations, he helps us gain a bigger picture than we otherwise would. It is not clear that the Kuhnian and Marxist approaches really work, since Gar did not in the end manage to develop and summarize what he thought they offered. But we can get some sense, and we learn from his perspective and his broad approach to history.

His dissertation advisor at Harvard, Everett Mendelsohn, captured Gar very well in his “Garland Allen: An Appreciation” in the *Journal of the History of Biology* (Mendelsohn 2016). As Everett explains there, “Garland Allen came of age, intellectually, professionally and politically during the two turbulent decades of the 1960s and 1970s.” Everett notes that Gar joined Martin Luther King’s march from Selma to Montgomery, Alabama in 1965. He joined a group of students on strike at Harvard to demonstrate against US involvement in the Vietnam War, including occupying administration buildings. This social engagement and activism led Gar to a strong sense of social justice. Gar opposed racism and other forms of bigotry, and he deplored the use of genetics as an attempt to justify actions against others. This sense of social good influenced his evolving scholarly approach.

This last book on which he was working also added something that was less central to his earlier scholarship. Gar wanted not just to acknowledge that science exists in a larger world, but also to connect the science more strongly with the social, political, and economic contexts. He talked about contexts, and he often wrote as if he were envisioning the science as a defined set of ideas and practices but embedded in a larger social environment. The science and the social environment remained separate for most of Gar’s writing. That social environment influenced what kinds of scientific questions were pursued, Gar thought, yet he was less clear whether context shaped the nature of the science itself. When he dug into issues of race and sexuality, for example, he saw claims that purported to be scientific as causing great social harm. Yet he was less clear whether the science itself was problematic or whether the problems lay with the ways science was used, or how the two interplayed and mutually influenced each other. I think he was still working this out in his own mind as he continued working on the later chapters of the book.

At the very end of the final chapter that he completed, Gar summarized his work in traditional historical terms of scientific ideas, how science works, and what transformations had occurred since Darwin:^10^

The central ideas of modern molecular biology have shown the same kind of unifying effect on the study of life as Darwin's did a century earlier. Yet the criteria of theory building and of seeking certain types of evidence were wholly different in the two periods. Molecular biology saw the culmination of a trend, first begun in the 1880s by Roux's *Entwicklungsmechanik*, of treating the organism in an experimental way, using the tools of physics and chemistry. Whereas Darwin's theory did not suggest any immediate ways in which it could be experimentally tested (in fact, it was thought that evolution was too slow to ever be observed in the laboratory), the basic tenets of the central dogma could be (and were!) almost immediately put to the test. Predictions from Darwin's theory could not be verified, whereas those from the central dogma could be easily verified (or contradicted, as the case may be). Thus, while both theories provided a focus for unifying a number of disparate areas of biology, they achieved this end by wholly different methodologies. It is this change in methodology which has characterized the growth of life sciences from the late nineteenth century through the present time.

We know that he did not want to stop there. I wish I knew what Gar had wanted to say in the final chapter that he did not write. His older daughter Tania Allen suggests that it helps to recall that Gar had an “utter commitment to knowing that revolution and change only emerged from differences in thinking and perspective. Throughout his entire career and life, he was encouraging people to expand their thinking, to be critical (about themselves, about him, about ideas) and believed that critical discourse was central to that change. I think he always encouraged that, no matter the subject and I have felt that in my own discussions with him throughout my life (even talking about pop culture -- we had a good argument about Eminem one time).” As Tania said to me, “the process of the book was really in sync with how his thinking was constantly evolving. The unfinished manuscript is a sort of ‘living document’ -- something that he would want others to build on, to borrow from, to argue against.”

And so we shall build on, borrow from, and argue against Gar’s ideas from this living document. Thank you, Gar. You were an excellent scholar, a wonderful friend and father and grandfather, and you will continue to inspire us.

You can read the manuscript as he left it. It resides with the Garland E. Allen Papers, located in the University Archives at Washington University in St. Louis, Missouri, though the papers have not yet been formally organized and described.^11^

## Footnotes

1 Garland Allen’s book in progress, provisionally entitled *From Darwin to the Double Helix: The Development of Genetics in its Intellectual, Social and Economic Context, 1880–1980*, is available by contacting Sonya Rooney, University Archivist, at spec@wustl.libanswers.com (hereafter, Allen, *Darwin to the Double Helix*).

2 Allen, *Darwin to the Double Helix,* Introduction*.*

3 Ibid.

4 Ibid.

5 Ibid.

6 I do not know which translation and edition of Marx Gar originally read. He had several versions in his various library collections.

7 Allen, *Darwin to the Double Helix,* Chapter 8.

8 Personal communication throughout February and March 2023. Mark Adams had a lively series of emails with Gar, discussing especially Adams’ work on Soviet science.

9 Allen, *Darwin to the Double Helix*, Chapter 1.

10 Allen, *Darwin to the Double Helix,* Chapter 16.

11 For questions about the collection or to access an electronic version of the book manuscript, you may contact Sonya Rooney, University Archivist, by emailing spec@wustl.libanswers.com.
